# Association of TSH Elevation with All-Cause Mortality in Elderly Patients with Chronic Kidney Disease

**DOI:** 10.1371/journal.pone.0168611

**Published:** 2017-01-03

**Authors:** Mei-hsing Chuang, Kuo-Meng Liao, Yao-Min Hung, Yi-Chang Chou, Pesus Chou

**Affiliations:** 1 Division of Family Medicine, Department of Community Medicine, Taipei City Hospital, Taipei, Taiwan; 2 Institute of Public Health and Community Medicine Research Center, National Yang-Ming University, Taipei, Taiwan; 3 Division of Endocrinology and Metabolism, Department of Internal Medicine, Taipei City Hospital, Taipei, Taiwan; 4 Department of Emergency Medicine, Kaohsiung Veterans General Hospital, Kaohsiung, Taiwan; 5 Department of Education and Research, Taipei City Hospital, Taipei, Taiwan; The University of Tokyo, JAPAN

## Abstract

Chronic kidney disease (CKD) is a widespread condition in the global population and is more common in the elderly. Thyroid-stimulating hormone (TSH) level increases with aging, and hypothyroidism is highly prevalent in CKD patients. However, the relationship between low thyroid function and mortality in CKD patients is unclear. Therefore, we conducted a retrospective cohort study to examine the relationship between TSH elevation and all-cause mortality in elderly patients with CKD. This retrospective cohort study included individuals ≥65 years old with CKD (n = 23,786) in Taipei City. Health examination data from 2005 to 2010 were provided by the Taipei Databank for Public Health Analysis. Subjects were categorized according to thyroid-stimulating hormone (TSH) level as follows: low normal (0.34<TSH<1.074 mIU/L), middle normal (1.074≤TSH≤2.46 mIU/L), high normal (2.46<TSH<5.2 mIU/L), elevated I (5.2≤TSH<10 mIU/L), and elevated II (TSH≥10 mIU/L). Risk of mortality was evaluated using a Cox proportional hazard regression model adjusted for sex, age, hypertension, diabetes mellitus, CKD stage, serum albumin, high-density lipoprotein cholesterol, uric acid, hemoglobin, body mass index, glutamic-pyruvic transaminase, smoking, alcohol consumption, and history of cardiovascular disease (coronary artery disease, congestive heart failure, cerebral vascular disease), history of cancer, and history of chronic obstructive pulmonary disease. Our results showed that compared to the reference group (middle normal TSH), the risk of all-cause mortality was increased in the elevated I group (hazard ratio [HR], 1.21; 95% confidence interval [CI], 1.02–1.45) and elevated II group (HR, 1.30; 95% CI, 1.00–1.69). We found a significant association between TSH elevation and all-cause mortality in this cohort of elderly persons with CKD. However, determining the benefit of treatment for moderately elevated TSH level (5.2–10 mIU/L) in elderly patients with CKD will require a well-designed randomized controlled trial.

## Introduction

Chronic kidney disease (CKD) is a common condition worldwide, with a prevalence ranging from 5.8% to 13.1% in different countries [1], and is particularly common in the elderly population. Aging, hypertension, diabetes, and cardiovascular disease (CVD) are important determinants of CKD [2], but some cases of CKD are of unknown etiology [2, 3]. A recent epidemiological study found a significant relationship between elevated thyroid-stimulating hormone (TSH) level and the development of CKD in elderly persons [4]. Other studies have shown that TSH level increases with aging in the general population [5, 6, 7, 8, 9], and hypothyroidism is prevalent among CKD patients [10, 11, 12]. However, the relationship between mild thyroid dysfunction and mortality is unclear.

Decreased thyroid function may be a protective mechanism to reduce protein catabolism [13]. Accordingly, several studies reported that elevated serum TSH level is associated with longevity [14, 15]. A large cohort study in Denmark also reported that subclinical hypothyroidism may be associated with a lower risk of all-cause mortality in individuals older than 65 years [16]. However, other studies have reported that both subclinical hypothyroidism (increased TSH level with normal free thyroxine level) and overt hypothyroidism are associated with a higher risk of all-cause and cardiovascular mortality in adults [17, 18, 19, 20, 21, 22]. Hypothyroidism was also reported to be associated with increased mortality in chronic dialysis patients [23, 24, 25, 26, 27, 28].

Low thyroid function is also associated with elevated plasma total cholesterol and low-density lipoprotein cholesterol [29, 30] and increased carotid intima-media thickness [31, 32, 33]. These findings suggest that abnormal thyroid function may increase the risk of CVD [34]. Patients on chronic dialysis may have impaired immune function, decreased antioxidant defense, accumulation of carcinogenic substances, and chronic infections and inflammation, suggesting that CKD patients may have a higher risk of cancer [35]. Consistent with this idea, an increased incidence of cancer has been observed in non-dialysis CKD patients [36] and chronic dialysis patients [37,38]. However, few studies have assessed the relationship between abnormally low thyroid function and mortality in elderly patients with CKD. Therefore, we conducted a retrospective cohort study to analyze the relationship between TSH elevation and all-cause mortality in elderly patients (≥65 years old) with CKD.

## Materials and Methods

### Patients and data source

The data used in this retrospective cohort study were provided by the Taipei Databank for Public Health Analysis, an official healthcare database in Taipei City. The Taipei City Government offers free annual health examinations for citizens aged ≥65 years in qualified hospitals. The database compiles longitudinal health examination data of these patients including demographic characteristics, medical history, medication history, alcohol consumption, smoking status, vital status, and laboratory results. The vital status and cause of death were matched with national death records. All identifying information associated with the data was removed before release.

We included elderly individuals who underwent health examinations from 2005 to 2010 and had prevalent CKD. Exclusion criteria were age <65 years old, receiving medication for thyroid disease, missing serum TSH data, missing body weight data, TSH ≤0.34 mIU/L, and only one check-up in 2010. The identification data of the participants were removed before we analyzed them. The study was approved by the institutional review board of Taipei City Hospital (No. CHIRB-1020410-E).

### Chronic kidney disease definition

In this study CKD was defined as estimated glomerular filtration rate (eGFR) <60 mL/min/1.73 m^2^ or eGFR ≥60 mL/min/1.73 m^2^ with proteinuria ≥1+. We estimated GFR levels using the 4-variable version of the Modification of Diet in Renal Disease equation as follows: eGFR (mL/min/1.73 m^2^) = 186 × serum creatinine ^-1.154^ × age ^-0.203^ × 0.742 (if female) [39]. Because we lacked urine albumin data, CKD stages were defined as follows: stage 1, eGFR ≥90 mL/min/1.73 m^2^ with positive urinary protein results; stage 2, eGFR 60–89 mL/min/1.73 m^2^ with positive urinary protein results; stage 3, eGFR 30–59 mL/min/1.73 m^2^; stage 4, eGFR 15–29 mL/min/1.73 m^2^; and stage 5, eGFR <15 mL/min/1.73 m^2^.

### Categorization of subjects according to TSH level

TSH levels were determined every other year using a third-generation assay. A total of 91,609 subjects had TSH data. We excluded individuals taking thyroid medication (n = 1,439) and those with TSH levels ≥99 mIU/L (n = 14) or 0 mIU/L (n = 92). Of the remaining 90,094 subjects, the 2.5th, 25th, 50th, 75th, and 97.5th percentiles for TSH level were 0.34, 1.074, 1.26, 2.46, and 6.45 mIU/L, respectively. The subjects were categorized according to baseline serum TSH level as follows: (1) low normal (0.34<TSH<1.074 mIU/L); (2) middle normal (1.074≤TSH≤2.46 mIU/L); (3) high normal (2.46<TSH<5.2 mIU/L); (4) elevated I (5.2≤TSH<10 mIU/L); or (5) elevated II (TSH ≥10 mIU/L) (mIU/L). The middle normal group served as the reference group.

### Possible confounders in the relationship between TSH level and mortality

Possible confounders include hypertension, diabetes mellitus (DM), history of CVD (coronary artery disease, congestive heart failure, cerebral vascular disease), history of cancer, history of chronic obstructive pulmonary disease (COPD), low serum albumin, dyslipidemia, hyperuricemia, abnormal liver function, anemia, low or high body mass index (BMI), smoking, and alcohol consumption. Hypertension was defined as systolic blood pressure ≥140 mmHg, diastolic blood pressure ≥90 mmHg, or use of anti-hypertensive medications or was determined based on medical history. Diabetes was defined as a fasting serum glucose ≥126 mg/dL or use of anti-diabetic medications, or was determined based on medical history. History of CVD, cancer, and COPD were determined by the patient’s medical history or known cause of death. Smoking status was categorized as never, occasional smoker, or frequent smoker. Alcohol consumption was categorized as never, occasional, or frequent.

### Ascertainment of all-cause, CVD, infectious disease, cancer, and respiratory disease deaths

The vital status of all subjects as of December 31, 2010 was ascertained from national death records. The underlying cause of death was coded according to the World Health Organization’s International Classification of Diseases, ninth revision (ICD-9) or tenth revision (ICD-10). The ICD-9 was used for records dating from 2006 to 2008, and the ICD-10 was used for records dating from 2009 to 2010. Cause of death included all causes (ICD-9: 001–998; ICD-10: A00–Z99), CVDs (ICD-9: 390–454; ICD-10: I00–I779), infectious diseases (ICD-9: 001–139, 460–490, 507, 567, 574, 576, 597, 599, 711; ICD-10: A00–B99, J159–J189, J860–J90, K63, K65, K75, K80, K81, N34, N45), cancers (ICD-9: 140–239; ICD-10: C00–C96, D00–D49), and respiratory tract diseases (ICD-9: codes 491–496, 500–508; ICD-10: J40–J849, J942–J984).

### Statistical analysis

Data were reported as mean ± standard deviation (SD) or n (%), as appropriate. Groups were compared by analysis of variance, followed by the Bonferroni correction. Differences in proportions were tested using the chi-square test. Survival time was calculated from the first examination (year) until death or censoring. Data were censored at the end of the follow-up period. Survival analyses were performed using the Cox proportional hazards model. The relative risk of mortality was determined using multivariate Cox regression analysis and presented as hazard ratio (HR) and 95% confidence interval (CI). A Cox proportional hazard model adjusted for hypertension, DM, history of CVD, history of cancer, history of COPD, serum albumin, CKD stages, high-density lipoprotein cholesterol (HDL-C), uric acid, glutamic-pyruvic transaminase (GPT), hemoglobin, BMI (kg/m^2^), history of smoking, and history of drinking. A p-value <0.05 was considered significant. Statistical analysis was performed using SAS 9.4 software (SAS Institute, Inc., Cary, NC, USA).

## Results

**Fig 1:**
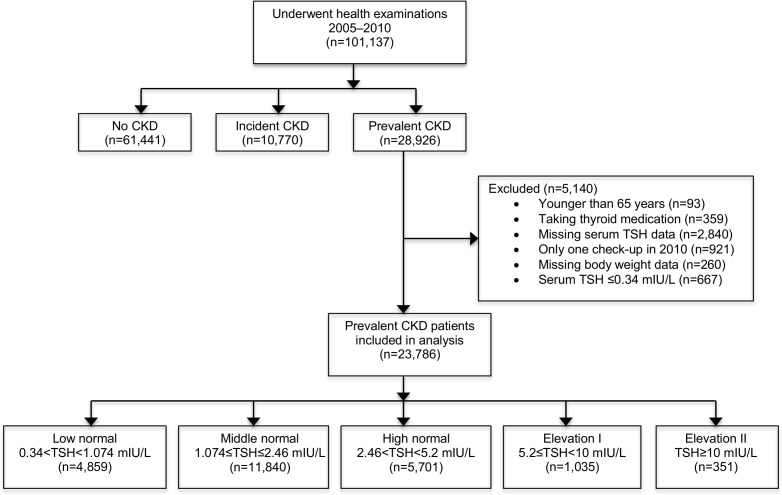
Flow chart of study subjects. Abbreviations: CKD, chronic kidney disease; TSH, thyroid-stimulating hormone.

Of the 101,137 elderly individuals who underwent health examinations from 2005 to 2010, 23,786 were included in the analysis (Fig 1).

The cohort included 10,466 women (44.0%), and the mean age was 77.25±6.89 years (Table 1). The 5-year study period yielded 107,346 person-years of follow-up, with a mean follow-up of 4.5±1.5 years. Categorization of the subjects according to TSH level resulted in 4,859 (20.4%) in the low normal group, 11,840 (49.8%) in the middle normal group, 5,701 (24.0%) in the high normal group, 1,035 (4.3%) in the elevated I group, and 351 (1.5%) in the elevated II group. These five groups were similar in terms of prevalence of hypertension, DM, history of cancer, and history of COPD. However, mean age and prevalence of CVD history were higher in the elevated II group compared with the other groups, and the proportion of women was higher in the two groups with elevated TSH levels.

**Table 1:** Baseline characteristics of elderly subjects with chronic kidney disease (CKD) according to thyroid-stimulating hormone (TSH) level.

	All subjects	Low normal (0.34<TSH<1.074 mIU/L)	Middle normal (1.074≤TSH≤2.46 mIU/L)	High normal (2.46<TSH<5.2 mIU/L)	Elevated I (5.2≤TSH<10 mIU/L)	Elevated II (TSH≥10 mIU/L)	p-value
Number (%)	23786 (100)	4859 (20.4)	11840 (49.8)	5701 (24.0.)	1035 (4.3)	351 (1.5)	
TSH, mIU/L	2.45±3.82	0.78±0.20	1.68±0.39	3.37±0.71	6.70±1.24	24.26±19.29	<0.01
Age, y	77.25±6.89	77.14±6.78	77.18±6.86	77.33±6.98	77.59±7.35	78.54±6.79	<0.01
Female, n (%)	10466 (44.0)	2179 (44.8)	4930 (41.6)	2636 (46.2)	539 (52.1)	182 (51.9)	<0.01
Comorbidities, n (%)							
Hypertension	12107 (50.9)	2462 (50.7)	6005 (50.7)	2927 (51.3)	543 (52.5)	170 (48.4)	0.63
Diabetes mellitus	5741 (24.1)	1139 (23.4)	2921 (24.7)	1366 (24.0)	243 (23.5)	72 (20.5)	0.20
History of cancer	1251 (5.3)	260 (5.4)	616 (5.2)	306 (5.4)	49 (4.7)	20 (5.7)	0.91
History of COPD	279 (1.2)	73 (1.5)	125 (1.1)	63 (1.1)	12 (1.2)	6 (1.7)	0.13
History of CVD	795 (3.3)	154 (3.2)	385 (3.3)	203 (3.6)	31 (3)	22 (6.2)	0.02
BMI, kg/m^2^	24.59±3.56	24.39±3.55	24.66±3.55	24.63±3.55	24.57±3.60	24.42±3.76	<0.01
CKD stage, n (%)							<0.01
Stage 1	532 (2.2)	136 (2.8)	269 (2.3)	108 (1.9)	16 (1.2)	3 (0.9)	
Stage 2	6334 (26.6)	1383 (28.5)	3194 (27.0)	1429 (25.1)	257 (24.8)	71 (20.2)	
Stage 3	15236 (64.1)	3054 (62.8)	7584 (64.1)	3674 (64.4)	6731 (65.0)	251 (71.5)	
Stage 4	1263 (5.3)	218 (4.5)	602 (5.1)	360 (6.3)	66 (6.4)	17 (4.8)	
Stage 5	421 (1.8)	68 (1.4)	1914 (1.6)	130 (2.3)	23 (2.2)	9 (2.6)	
Laboratory test results							
Serum albumin, g/dL	4.28±0.34	4.29±0.34	4.29±0.34	4.27±0.36	4.27±0.35	4.23±0.37	<0.01
HDL-C, mg/dL	50.18±13.63	51.16±13.75	49.92±13.52	49.88±13.68	49.83±13.92	51.21±13.69	<0.01
Uric acid, mg/dL	6.62±1.76	6.55±1.75	6.62±1.75	6.70±1.77	6.57±1.75	6.55±1.89	<0.01
GPT, U/L	22.76±18.89	22.11±17.66	22.71±18.16	23.19±19.92	23.26±25.14	24.83±20.57	<0.01
Hemoglobin, g/dL	13.13±1.67	13.19±1.65	13.20±1.66	13.01±1.68	12.91±1.71	12.69±1.63	<0.01
Smoking status, n (%)							<0.01
Non-smoker	21446 (90.2)	4257 (87.6)	10639 (89.9)	5247 (92.0)	974 (94.1)	329 (93.73)	
Occasional smoker	2340 (9.8)	602 (12.4)	1201 (10.1)	454 (8.0)	61 (5.9)	22 (6.3)	
Alcohol consumption, n (%)							
Non-drinker	19965 (83.9)	4052 (83.4)	9899 (83.6)	4799 (84.4)	907 (87.6)	308 (87.8)	<0.01
Occasional	3418 (14.4)	721 (14.9)	1752 (14.8)	792 (13.9)	117 (11.3)	36 (10.3)	
Frequent	403 (1.7)	86 (1.8)	189 (1.6)	110 (1.9)	11 (1.06)	7 (2.0)	

Results are presented as n (%) or (mean±SD).

Abbreviations: BMI, body mass index; COPD, chronic obstructive pulmonary disease; CVD, cardiovascular disease; GPT, glutamic-pyruvic transaminase; HDL-C, high-density lipoprotein cholesterol; SD, standard deviation

Results of post hoc analysis with the Bonferroni correction were as follows. The low normal TSH group had a significantly lower mean BMI than the middle normal and high normal groups. In addition, the low normal group had a significantly higher mean HDL-C level compared with the middle normal, high normal, and elevated I groups. The high normal group had significantly higher mean uric acid and GPT levels compared with the low normal group. The elevated I group had a significantly lower mean hemoglobin compared with the low normal and middle normal groups. The elevated II group had a significantly higher prevalence of CKD stage 3 compared with the other four groups and significantly lower mean serum albumin compared with the low normal and middle normal groups. In addition, the elevated II group had a significantly lower mean hemoglobin level compared with the low normal, middle normal, and high normal groups.

During the 5-year follow-up, 3,035 (12.8%) deaths occurred, including 864 (28.5%) deaths due to cancers, 795 (26.2%) deaths due to CVD, 359 (11.8%) deaths due to infectious diseases, and 139 (4.6%) deaths due to respiratory tract diseases. The adjusted HRs and 95% CIs for death are shown in Table 2. TSH elevation between 5.2 and 10 mIU/L (elevated I group) was associated with increased mortality risk compared to TSH level between 1.074 and 2.46 mIU/L (middle normal group) (HR, 1.21; 95% CI, 1.02–1.45). TSH elevation ≥10 mIU/L appeared to be associated with an increased mortality risk compared to the middle normal TSH level (HR, 1.30; 95% CI, 1.00–1.69).

**Table 2:** Multivariate Cox regression analysis of potential baseline predictors of all-cause mortality.

Variables	HR	95% CI
Age, years	1.04	1.03–1.04
Sex		
Male	1	
Female	0.72	0.66–0.79
TSH level		
Low normal (0.34<TSH<1.074 mIU/L)	1.06	0.96–1.16
Middle normal (1.074≤TSH≤2.46 mIU/L)	1	
High normal (2.46<TSH<5.2 mIU/L)	0.97	0.89–1.06
Elevated I (5.2≤TSH<10 mIU/L)	1.21	1.02–1.45
Elevated II (TSH≥10 mIU/L)	1.30	1.00–1.69
Hypertension	1.81	1.67–1.95
Diabetes mellitus	1.47	1.36–1.60
History of cardiovascular disease	22.40	20.35–24.67
History of cancer	14.17	12.96–15.49
History of COPD	5.59	4.60–6.79
Chronic kidney disease		
Stage 1–2	1	
Stage 3	1.18	1.07–1.30
Stage 4	1.54	1.33–1.80
Stage 5	1.50	1.22–1.85
Serum albumin, g/dL	0.51	0.46–0.56
HDL-C, mg/dL	0.994	0.991–0.997
BMI, kg/m^2^	0.92	0.91–0.93
Uric acid, mg/dL	1.04	1.02–1.06
GPT, U/L	1.003	1.001–1.004
Hemoglobin, g/dL	0.91	0.89–0.93
Smoking status		
Non-smoker	1	
Frequent smoker	1.41	1.26–1.57
Alcohol consumption		
Non-drinker	1	
Occasional drinker	0.88	0.78–0.99
Frequent drinker	1.17	0.90–1.52

Abbreviations: BMI, body mass index; COPD, chronic obstructive pulmonary disease; GPT, glutamic-pyruvic transaminase; HDL-C, high-density lipoprotein cholesterol; HR, hazard ratio; TSH, thyroid-stimulating hormone.

With CVD mortality or malignancy mortality as the dependent variables, the risk of death was not significantly greater when TSH level was elevated. No interactions were found between TSH level and CKD, treating CKD as a dichotomous variable (stages 1/2 vs. stages 3–5). Interactions between TSH level and sex and age (<85 or ≥85 years) were not significant.

## Discussion

In this retrospective observational study, we found that that TSH elevation, which suggests low thyroid function, was associated with an increased mortality risk in elderly patients with CKD in Taipei City. Serum TSH level increases slowly with age in the elderly population, with the normal range in adults older than 70 years old reported as 0.62–6.15 mIU/L [40] and 0.47–7.11 mIU/L [41]. In our study of 90,094 subjects older than 65 years who had CKD, the 2.5th, 25th, 50th, 75th, and 97.5th percentiles of TSH level were 0.34, 1.074, 1.26, 2.46, and 6.45 mIU/L, respectively. Treatment for subclinical hypothyroidism (TSH level between 5 and10 mIU/L) is not well established [42]. In this study we classified subjects into five groups based on TSH level, using a cutoff value of 5.2 mIU/L to define TSH elevation.

Both subclinical and overt hypothyroidism are common in CKD patients [5–9]. For example, decreased iodine excretion due to impaired glomerular filtration can lead to elevated serum iodine concentration, thereby blocking thyroid hormone production (Wolff–Chaikoff effect) [43]. In addition, low thyroid function appears to be a protective mechanism to save energy and is associated with a longer life span [14,15]. However, hypothyroidism is also associated with several risk factors for CVD such as dyslipidemia, systolic and diastolic hypertension, atherosclerosis, ventricular arrhythmia, and decreased endothelial vasodilation [29–34].

Our results are consistent with previous studies reporting that subclinical and overt hypothyroidism are associated with an increased risk of mortality in adults. In a meta-analysis of 11 cohort studies with 55,287 participants, subclinical hypothyroidism (TSH concentration ≥10 mIU/L) was associated with an increased risk of coronary heart disease (CHD) events and CHD mortality in individuals younger than 65 years [18]. However, a large Danish study found that subclinical hypothyroidism (TSH 5–10 mIU/L) was associated with a decreased risk of all-cause mortality only in individuals older than 65 years [16]. Age-dependent susceptibilities to mild hypothyroidism may explain this discrepancy [44,45]. In our cohort, moderately elevated TSH level (5.2≤TSH<10 mIU/L) was significantly associated with all-cause mortality. The nonsignificant association between TSH level ≥10 mIU/L and all-cause mortality may have been due to the relatively small number of subjects in that TSH group (n = 351, 1.5%). Our finding that thyroid dysfunction was not significantly associated with mortality in subjects older than 85 years could potentially be explained by the energy-saving benefit of mild hypothyroidism in this age group [44,45]. However, subjects older than 85 years with elevated TSH (≥5.2 mIU/L) did not have a lower risk of death.

In our study, the HRs for history of CVD and history of cancer were high, which is consistent with the results of previous studies. For example, CVDs were identified as the most common causes of death for end-stage renal disease patients by the US Renal Data System [46]. A more recent study in the US found that CVDs and cancer were the leading causes of death in CKD patients, with more cancer deaths than CVD deaths in patients in the earlier stages of kidney disease [36]. Similarly, a recent study in Hong Kong reported a higher incidence of cancer in chronic dialysis patients compared with the age- and sex-matched general population [38].

In our study, 3,035 (12.8%) of the subjects died during the 5-year study period: 864 (28.5%) deaths were cancer-related, and 795 (26.2%) were CVD-related. We found that the risk of CVD death was not significantly greater in subjects with elevated TSH levels. In addition, only 1.8% of the elderly subjects had stage 5 CKD (Table 1). Thus, a longer follow-up may be needed to better understand the relationship between low thyroid function and cause of death among elderly individuals with CKD.

The main strength of this research study was its large sample size, which allowed us to perform stratified analyses. Second, we collected a significant amount of clinical data to adjust for multiple confounders. Third, we were able to verify cause of death. However, several limitations of the study must be mentioned. First, of the thyroid hormones, only TSH level was measured. The lack of free thyroxine data prevented us from differentiating between subclinical hypothyroidism and overt hypothyroidism. Second, the elderly subjects who underwent annual health examinations may not truly represent the general population, limiting the generalizability of our results to all elderly patients with CKD. Third, because of the observational nature of the data, there may be unobserved variables that influenced survival. Therefore, we are unable to infer a causal relationship between hypothyroidism and patient outcome.

In conclusion, we found a significant association between TSH elevation and mortality in elderly patients with CKD. An increased death risk was observed in individuals with TSH level higher than 5.2 mIU/L. This finding suggests that periodic evaluation of TSH level in elderly CKD patients may be useful to detect abnormal thyroid function. However, a well-designed randomized controlled trial is needed to evaluate the benefit of treating TSH elevation in elderly patients with CKD. Further studies to investigate the need for cancer screening in CKD patients may also be necessary.
